# 129. Safety and Cost-effectiveness Analysis Outpatient Continuous Parenteral Antibiotics via a Disposable Elastomeric Pump at Two County Hospitals in Houston, Texas

**DOI:** 10.1093/ofid/ofab466.331

**Published:** 2021-12-04

**Authors:** Aishwarya Rao, Sam Karimaghaei, Juliet Chijioke, Kristin Constance, Natalie Finch, Nigo Masayuki

**Affiliations:** 1 McGovern Medical School, The University of Texas Health Science Center at Houston, Houston, Texas; 2 Harris Health System, Houston, Texas; 3 University of Texas Medical Center at Houston, Houston, Texas; 4 University of Texas in Houston, Houston, TX

## Abstract

**Background:**

Outpatient parenteral antibiotic therapy (OPAT) is a therapeutic option for patients who require longer intravenous (IV) antimicrobial courses, yet do not need to remain hospitalized. HarrisHealth system OPAT programs implement a disposable elastomeric continuous infusion pump (eCIP) for IV antibiotics. Here we report the clinic-demographic features, outcomes of a cohort of patients receiving OPAT via eCIP (OPAT-eCIP), as well as the cost-effectiveness of OPAT in comparison to standard inpatient care.

**Methods:**

We retrospectively obtained the clinic-demographic characteristics and outcomes of 91 patients discharged from HarrisHealth-affiliated hospitals from December 2018 to February 2021 who underwent OPAT-eCIP. We then compared the total costs associated with home OPAT-eCIP care with that of an equivalent of inpatient IV antimicrobial treatment based on previous studies.

**Results:**

We identified 481 total OPAT patients; 91 (18.9%) received intravenous antibiotics via eCIP, with two initiating therapy outpatient. In total, 1925 days of IV antimicrobial therapy were administered outpatient by OPAT-eCIP, with a median treatment course of 12 days. Eighty-three (92.2%) patients completed their antimicrobial course, with 85 (93.4%) cured of respective infections (Table 1). Antimicrobial-associated adverse events and PICC line associated complications were 6.6% and 14.3% respectively. 30-day hospital readmission rates were under 10% with 21 patients (23.1%, 28 total visits) presenting to the emergency room over the course of IV therapy. Estimated costs of OPAT-eCIP care over the study period ranged from &417,000-&576,750 with costs of equivalent inpatient care estimated at &2,945,250 to &3,927,000; estimated overall cost savings of OPAT-eCIP were &2,368,500 to &3,509,900 (Table 2).

Table 1. Characteristics and Outcomes of Patients Receiving Continuous IV Antibiotics via Disposable Elastomeric Pump

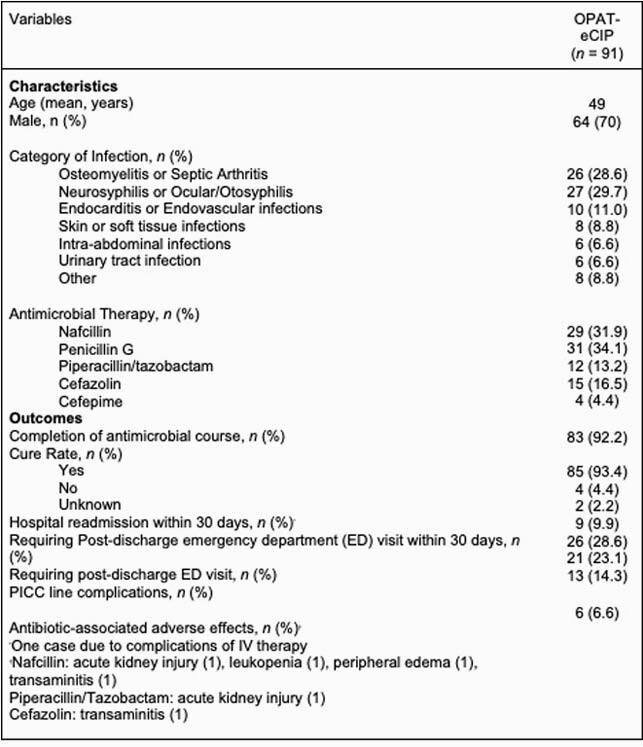

Table 2. Cost Analysis Comparison of OPAT-eCIP therapy versus inpatient antimicrobial therapy in patients from December 2018 from February 2021

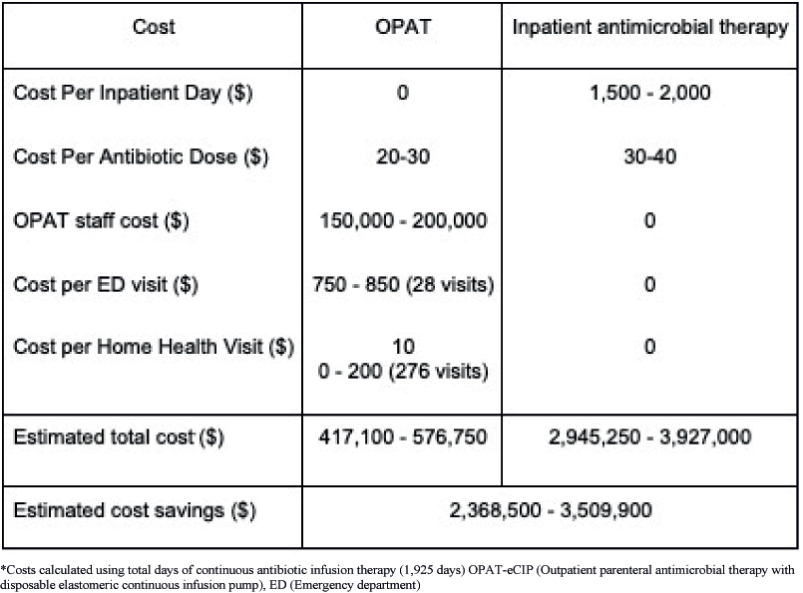

**Conclusion:**

OPAT-eCIP therapy in a cohort of patients was highly effective and well-tolerated. While ED visit frequency indicates the necessity of close patient monitoring, low 30-day hospital readmission rates were encouraging. Along with the above, the significant cost savings demonstrated when compared with standard inpatient antimicrobial therapy suggest that OPAT-eCIP should be increasingly utilized as an effective therapeutic option.

**Disclosures:**

**All Authors**: No reported disclosures

